# Culture on a native bone marrow-derived extracellular matrix restores the pancreatic islet basement membrane, preserves islet function, and attenuates islet immunogenicity

**DOI:** 10.1096/fj.201902893R

**Published:** 2020-04-19

**Authors:** Hanzhou Wang, Shengxian Li, Qiuxia Dai, Aaron Gonzalez, Olivia N. Tran, Haiyan Sun, Ralph A. DeFronzo, David D. Dean, Chih-Ko Yeh, Xiao-Dong Chen

**Affiliations:** 1 Department of Comprehensive Dentistry, University of Texas Health Science Center at San Antonio, San Antonio, TX, USA; 2 Department of Endocrinology, Renji Hospital, School of Medicine, Shanghai Jiaotong University, Shanghai, People’s Republic of China; 3 Department of Biomedical Engineering, University of Texas at San Antonio, San Antonio, TX, USA; 4 Department of Stomatology, Affiliated Hospital of the Academy of Military Medical Sciences, Beijing, People’s Republic of China; 5 Diabetes Division, Department of Medicine, University of Texas Health Science Center at San Antonio, San Antonio, TX, USA; 6 Department of Veterans Affairs, South Texas Veterans Health Care System, San Antonio, TX, USA

**Keywords:** extracellular matrix, immune tolerance, insulin secretion, pancreatic islets, vascular endothelial cells

## Abstract

Islet transplantation in man is limited by multiple factors including islet availability, islet cell damage caused by collagenase during isolation, maintenance of islet function between isolation and transplantation, and allograft rejection. In this study, we describe a new approach for preparing islets that enhances islet function in vitro and reduces immunogenicity. The approach involves culture on native decellularized 3D bone marrow-derived extracellular matrix (3D-ECM), which contains many of the matrix components present in pancreas, prior to islet transplantation. Compared to islets cultured on tissue culture plastic (TCP), islets cultured on 3D-ECM exhibited greater attachment, higher survival rate, increased insulin content, and enhanced glucose-stimulated insulin secretion. In addition, culture of islets on 3D-ECM promoted recovery of vascular endothelial cells within the islets and restored basement membrane-related proteins (eg, fibronectin and collagen type VI). More interestingly, culture on 3D-ECM also selectively decontaminated islets of “passenger” cells (co-isolated with the islets) and restored basement membrane-associated type VI collagen, which were associated with an attenuation in islet immunogenicity. These results demonstrate that this novel approach has promise for overcoming two major issues in human islet transplantation: (a) poor yield of islets from donated pancreas tissue and (b) the need for life-long immunosuppression.

## 1 ∣ INTRODUCTION

Type 1 diabetes (T1D) is an autoimmune disease that leads to pancreatic β-cell destruction and insulinopenia. For many years, allogeneic islet transplantation has been the focus of intense investigation as an insulin replacement therapy. This requires isolating pancreatic islets from two to four cadaveric donors and infusing them into the portal vein of the recipient.^1^ Although insulin independence is achieved initially, by 5 years posttransplantation the number of patients still capable of maintaining normoglycemia declines to about 10%.^2,3^ This approach has multiple limitations including lack of donor tissue and limited numbers of islets per patient,^2,3^ damage to islets during isolation resulting in islet cell apoptosis (anoikis) and increased immunogenicity,^6,7^ and the need for lifelong immunosuppression to prevent rejection.^2^ These issues must be resolved before islet transplantation can be a viable therapy for T1D.^2,3^

At present, the standard procedure for isolating islets involves collagenase digestion of donor pancreas tissue, islet purification, and then, maintenance in culture until the transplant is performed.^4^ During this process, the islet basement membrane and associated extracellular matrix (ECM) proteins are damaged.^4^ Prior studies have clearly demonstrated that the basement membrane serves as a barrier that prevents immune cell attack and promotes the retention of islet architecture.^5,6^ Once the basement membrane is lost or damaged, islet cell anoikis and immunogenicity are increased, leading to increased risk of islet allograft rejection.^6,7^

It is well-accepted that in vitro culture of islets significantly diminishes the number of various contaminating or “passenger” cells and prolongs islet allograft survival in vivo. However, when islets are cultured on TCP, the individual islets, which are normally spherical in shape, become flattened, and lose their function,^8^ indicating that critical factor(s), present in the pancreatic microenvironment, are missing in this culture system. Islets in vivo are surrounded by an ECM, which forms a unique microenvironment (niche) that provides critical architectural, biochemical, and physical cues for maintaining their differentiated function. Studies using a variety of synthetic materials (eg, polymeric biomaterials), purified collagens, fibronectin or laminin, or mixtures of ECM components (eg, Matrigel) have been shown to extend β-cell survival and function better than TCP.^9-11^ However, it is unlikely that the native ECM can be replicated using these simple synthetic or purified/recombinant components. It would be ideal if an authentic pancreatic microenvironment (niche) could be replicated ex vivo. However, preparing a pancreatic-derived ECM is very challenging because of difficulties in obtaining autologous/allogeneic pancreatic cells for producing the ECM and identifying the culture conditions needed.

Previously, we described a 3D culture system, established using cell-free native ECM generated by bone marrow stromal cells (3D-ECM), which enhanced both human and mouse MSC attachment, proliferation, and retention of “sternness”,^12,13^ and restored the quantity and quality of aging MSCs.^14,15^ This native ECM has a unique 3D architecture composed of collagens (types I, III, XII, and VI), fibronectin, small leucine-rich proteoglycans, and basement membrane components (perlecan and laminin).^12,13,16^ Since serendipitously bone marrow stromal cells synthesize a number of critical ECM components, previously identified in islets,^9^ we propose to use this bone marrow-derived ECM as a *surrogate* native pancreatic ECM. We hypothesize that culture of pancreatic islets on this 3D-ECM will promote pancreatic islet attachment and viability, maintain biological function, and restore the vascular basement membrane, while reducing islet immunogenicity. If the results confirm our hypothesis, they will provide the information necessary to begin identifying a combination of key effective ECM components necessary to preserve islet function.

## 2 ∣ MATERIALS AND METHODS

### 2.1 ∣ Experimental animals

Inbred male Lewis, Wistar-Furth, and Fischer 344 rats (220-350 g) were purchased from Envigo (Indianapolis, IN, USA) and used as a source of pancreatic islets, splenocytes, and bone marrow. Rats were fed standard rodent chow and water ad libitum and housed in an AAALAC-accredited vivarium. All use of the animals complied with the ARRIVE guidelines and all procedures were approved by the IACUC at UTHSCSA.

A rat model was chosen for these studies due to its cost, availability of genetic strains, and large volume of prior work, including pioneering islet transplantation studies.^8,17,18^ However, there are many differences between rodent and human islets and species-specific differences in immune systems.^19-21^ The rat model is very useful for laying the foundation of subsequent studies in higher order mammals [eg, canine or porcine and nonhuman primates^20^].

### 2.2 ∣ Preparation of 3D-ECM

Rat 3D-ECM plates were produced as previously described.^12,13^ Briefly, BM-MSCs were seeded onto TCP plates and cultured for 7 days in “growth media.” On day 7, ascorbic acid (50 μmol/L) was added to the media and culture continued for an additional 8 days. After decellularization, the ECM was washed with PBS, followed by sterile distilled water, and then, used in the experiments or dried before storing at 4°C. If dried, the ECM was re-hydrated with PBS for 1 hour at 37°C before use. For experiments using human 3D-ECM, plates were obtained from StemBioSys, Inc (San Antonio, TX, USA) (www.stembiosys.com).

### 2.3 ∣ Pancreatic islet isolation

Rat pancreatic islets were isolated, as previously described,^22^ by perfusion of 10 mL of collagenase P (1 mg/mL, Sigma-Aldrich, St. Louis, MO, USA) into the pancreas, surgical removal of the organ, followed by incubation at 37°C for 13 minutes. Subsequently, the islets were collected/purified on a Histopaque-1077 gradient and handpicked under a dissecting microscope. The purified islets were cultured on standard TCP plates (uncoated or coated with 3D-ECM or fibronectin) in RPMI-1640 media supplemented with 10% of heat-inactivated FBS and 1% of penicillin-streptomycin for 7 days. Depending on the experiment, collagenase II (Worthington Biochemical Corp., Lakewood, NJ, USA, 400 IU/mL) or 20 mmol/L EDTA was used to detach islets from the culture surface.

### 2.4 ∣ Pancreatic islet adhesion and viability assays

After varying times in culture, adherent and nonadherent islets were separated by gently transferring unattached islets to a new culture plate. The number of nonadherent and adherent islets was then determined visually using a dissecting microscope.

Islet viability was evaluated after staining with acridine orange (AO) and propidium iodide (PI) as described.^23^ Isolated islets were incubated for 10 minutes in Dulbecco’s PBS containing AO (0.67 μmol/L) and PI (75 μmol/L), and then, examined using fluorescence microscopy.

### 2.5 ∣ Glucose-stimulated insulin secretion (GSIS) assay

GSIS assays were performed as previously described.^24^ Briefly, a standardized number of islets (typically 20/well) were sequentially treated for 60 minutes at 37°C with 5.6 mmol/L glucose (all dissolved in KRBH-BSA buffer (115 mmol/L NaCl, 24 mmol/L NaHCO_3_, 5 mmol/L KCl, 1 mmol/L MgCl_2_, 1 mmol/L CaCl_2_, 20 mmol/L HEPES, 0.5% of BSA, pH = 7.4), 16.7 mmol/L glucose, 5.6 mmol/L glucose, and 30 mM KCl (to promote exocytosis of any remaining secretion-ready insulin granules).^25,26^ After each treatment, conditioned media were collected and insulin quantitated using a rat insulin ELISA (Crystal Chem USA, Elk Grove Village, IL, USA).

### 2.6 ∣ Mixed lymphocyte-islet coculture (MLIC) assay

MLIC assays were performed as previously described.^27^ Before assay, rat islets were treated for 60 minutes with 50 μg/mL of mitomycin C (Sigma-Aldrich, St. Louis, MO, USA) to suppress islet cell proliferation, and then, plated (30 islets/well) into 96 well plates. Rat splenocytes (2 × 10^5^ cells/well) were then added and the cocultures incubated for 4 days. Phytohemagglutinin (PHA) and splenocytes from other rat strains were used as positive controls. Splenocyte proliferation was assessed by use of a bromodeoxyuridine (BrdU) ELISA (Sigma-Aldrich, St. Louis, MO, USA).

### 2.7 ∣ Immunofluorescence (IF) microscopy

Pancreatic tissue or isolated islets were embedded in Tissue-Tek OCT Compound (VWR, Missouri City, TX, USA), and then, snap-frozen. Frozen sections (10 μm) were cut, placed on Superfrost glass slides, and then, stored at −80°C. For immunostaining, sections were fixed in methanol, followed by overnight incubation with primary antibodies raised against insulin (Santa Cruz Biotechnology, Dallas, TX, USA; 1:200 dilution), collagen IV, VI, fibronectin (Abcam, Cambridge, MA, USA; 1:200 dilution), and CD31 (R&D Systems, Minneapolis, MN, USA; 1:100 dilution). Alexa Fluor 647 conjugated secondary antibodies (Abcam; 1:500 dilution) were used for detection.

For immunofluorescence of paraffin embedded rat islets, blocks were cut into 4 μm sections, deparaffinized, rehydrated, and subjected to antigen retrieval with citrate buffer using a microwave oven on high power. Sections were immunostained with anti-insulin (see above) primary antibody followed by Alexa Fluor 488 conjugated antibody (Invitrogen, Carlsbad, CA, USA; 1:1000 dilution).

Both frozen and paraffin sections were mounted in Fluoroshield mounting media containing DAPI (Sigma-Aldrich, St. Louis, MO, USA). Images were captured using an Olympus IX73 fluorescence microscope or Zeiss LSM 710 confocal microscope.

### 2.8 ∣ Transmission electron microscopy (TEM)

For TEM, islets were fixed for 1 hour with 2% of glutaraldehyde in 0.1 mol/L sodium cacodylate buffer (pH 7.2), and then, transferred to 0.1 mol/L cacodylate buffer. Samples were then dehydrated, embedded in Epon resin (Polysciences, Inc., Warrington, PA, USA), sectioned, stained with uranyl acetate and lead citrate, and then, examined using a Jeol 1230 TEM (Jeol USA, Peabody, MA, USA). Secretory granules were identified, counted, and subjected to size analysis using ImageJ (Fiji) software (https://imagej.net/ImageJ).^8,28,29^

### 2.9 ∣ Coculture of human macrophage-like U937 cells with rat islets

Human U937 cells (CRL-1593.2) were purchased from the American Type Culture Collection (ATCC, Manassas, VA, USA) and cultured for 48 hours in RPMI 1640 medium supplemented with 10% of FBS and 10 ng/mL of phorbol-12-myristate-13-acetate (PMA) followed by 24 hours of rest to attain full differentiation.

Differentiated U937 cells were cocultured with freshly isolated islets or islets that had been precultured for 7 days on TCP or 3D-ECM and released from the culture surface with 20 mM/L EDTA. During coculture for 48 hours, 100 ng/mL of lipopolysaccharide (LPS) (a known activator of macrophages) or vehicle was added. At the end of coculture, islets were separated from the adherent macrophages to assess changes in pro-inflammatory (IL-1β, IL-6, and TNF-α) and anti-inflammatory cytokine (IL-10) transcripts (see RNA isolation below).

### 2.10 ∣ RNA isolation and real-time PCR

Total RNA was extracted from the macrophages using Trizol reagent (Invitrogen, Carlsbad, CA, USA). RNA (1 μg) was reverse transcribed using a High Capacity cDNA Reverse Transcription Kit (Applied Biosystems, Foster City, CA, USA). The transcripts of interest, including the housekeeping gene (GAPDH), were amplified from the cDNA using SYBR Green PCR assay. Amplification and detection were carried out using an ABI Prism 7900HT Sequence Detection System. Changes in gene expression were calculated using the delta delta Ct method with GAPDH as housekeeping gene. Primers were designed using Primer-BLAST (https://www.ncbi.nlm.nih.gov/tools/primer-blast/) and synthesized by Sigma-Aldrich (St. Louis, MO, USA):

**Table:** 

GAPDH	Forward: 5′-CCATCAATGACCCCTTCATTG-3′
	Reverse: 5′- GACGGTGCCATGGAATTTG-3′
IL-6	Forward: 5′-CAATGAGGAGACTTGCCTGGT-3′
	Reverse: 5′-CACAGCTCTGGCTTGTTCCT-3′
IL-1β	Forward: 5′-AGAAGTACCTGAGCTCGCCA-3′
	Reverse: 5′-CTGGAAGGAGCACTTCATCTG-3′
TNF-α	Forward: 5′-GCCTCTTCTCCTTCCTGATCG-3′
	Reverse: 5′-GAAGATGATCTGACTGCCTGGG-3′
IL-10	Forward: 5′-TACGGCGCTGTCATCGATTT-3′
	Reverse: 5′-ACTCATGGCTTTGTAGATGCCT-3′

### 2.11 ∣ Statistical analysis

All experiments were repeated a minimum of three times with replicates of 3 to 7 (actual number is specified in each figure legend). The data presented in the figures include all the data collected in one representative experiment and are the mean ± SEM Assignment of samples to either control or experimental groups was random; islets for a particular experiment were prepared from multiple donors, and then, pooled before random allocation to control or experimental groups. Statistical analyses were performed using GraphPad Prism software (version 7.0) (La Jolla, CA, USA). One-way or Two-way analysis of variance (ANOVA), followed by post hoc testing or student *t* tests (with Bonferroni correction), was utilized. *P* values ≤ .05 were considered significant.

### 2.12 ∣ Data and resource availability

The datasets generated and/or analyzed in this study are available from the corresponding author by request. All applicable resources are available for purchase from the indicated commercial entities; no new resources were generated or analyzed during the current study.

## 3 ∣ RESULTS

### 3.1 ∣ Culture on 3D ECM enhanced islet adhesion and promoted viability

**FIGURE 1:**
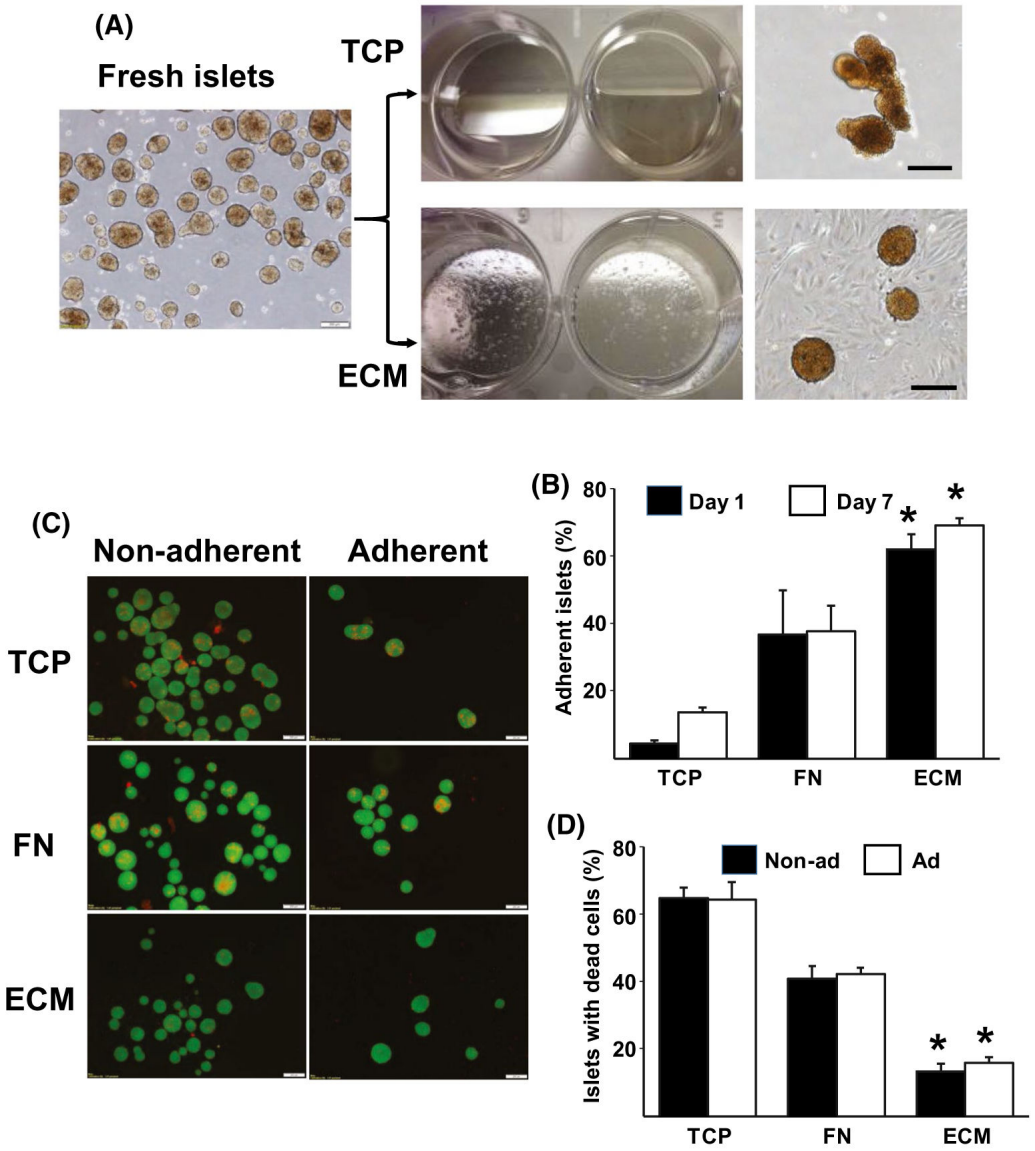
3D ECM enhanced islet adhesion and promoted viability. Freshly isolated rat islets [200 IEQ/cm^2^)] were cultured for 1 or 7 days on untreated TCP or TCP coated with fibronectin (FN) or 3D ECM. A, Phase contrast photomicrographs of islets cultured on either untreated TCP or TCP coated with 3D ECM for 7 days. Bar = 100 μm. B, Percentage of adherent islets after culture for 1 or 7 days on the three culture surfaces. The number of adherent and nonadherent islets were counted using phase contrast microscopy and the percent adherent calculated (mean ± SEM, n = 3/group), *P < .004 versus TCP or FN. C, After 7 days in culture on the three culture surfaces, islets were stained with AO (live cells, green) and PI (dead cells, red), and then, examined by fluorescence microscopy. Bar = 200 μm. D, After staining with AO/PI, the percent of islets containing dead cells was quantified (mean ± SEM, n = 3/group),*P = .008 versus TCP or FN. Data were analyzed using two-way ANOVA with Bonferroni correction

Freshly isolated islets were seeded directly onto uncoated TCP or TCP coated with fibronectin or 3D-ECM and cultured for 1 or 7 days. After 7 days, islets cultured on TCP were observed to form aggregates and not adhere well to the culture surface (Figure 1A, upper panel). In contrast, islets cultured on 3D-ECM were evenly distributed without forming aggregates. Interestingly, islets cultured on the 3D-ECM not only adhered better than on TCP, but also numerous fibroblast-like cells were found growing on the matrix (Figure 1A, lower panel), suggesting that during culture “passenger” cells migrated off the islets.

Nonadherent and adherent islets were collected after 1 or 7 days in culture on the various substrates and counted and islet viability was determined histochemically using AO and PI. After 1 and 7 days in culture on 3D-ECM, over 60% of the islets were found to have adhered to the culture surface, while far less adhered to TCP (<5%-10%) (Figure 1B). Moreover, both adherent and nonadherent islets maintained on 3D-ECM contained fewer dead cells than those maintained on TCP (Figure 1C,D). Since fibronectin has been reported to improve islet attachment and viability,^30,31^ we coated this matrix molecule onto TCP for comparison purposes and found that it improved islet attachment and viability better than TCP, but not as well as 3D-ECM.

### 3.2 ∣ Secretory granule number and size were increased in islets maintained on 3D ECM

**FIGURE 2:**
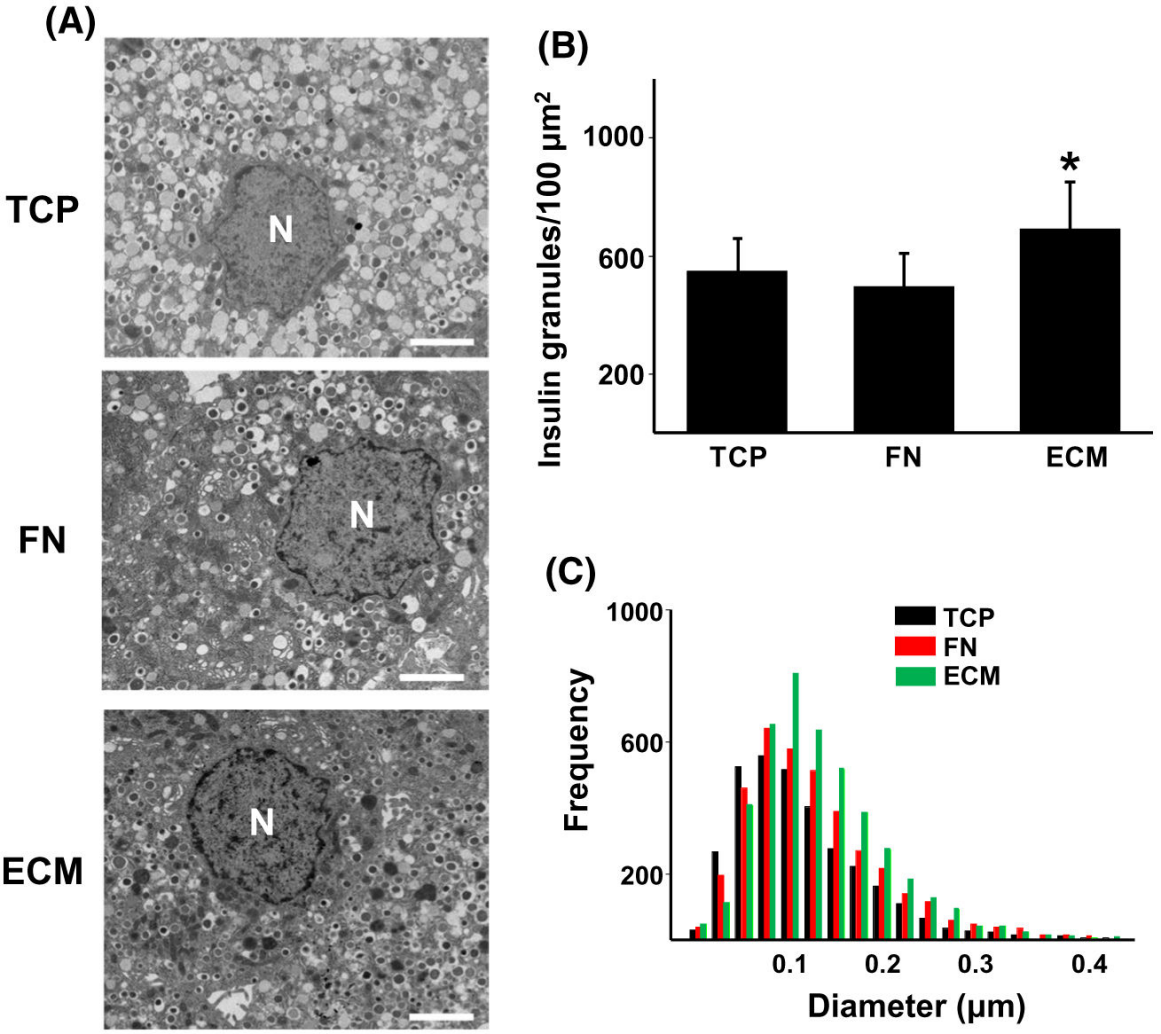
Secretory granule number and size were increased in islets maintained on 3D ECM. A, TEM photomicrographs of rat islets after culture for 7 days on untreated TCP and TCP coated with FN or 3D ECM. Numerous insulin-containing secretory granules, with densely stained cores, can be seen in the cytoplasm, especially in islets cultured on ECM *N*: cell nucleus. Bar = 2 μm. B, Morphometric analysis was used to determine the total number of insulin-containing secretory granules (normalized to an area of 100 μm^2^) in islets cultured on the three different substrates. The data are the mean ± SEM, n = 7 fields/group, *P = .04 versus TCP or FN. C, Morphometric analysis was used to prepare a frequency size distribution of the insulin-containing secretory granules on the three different substrates. Data were analyzed using one-way ANOVA with Bonferroni correction

To compare the effect of culture substrate on islet ultrastructure, morphometric analysis of TEM photomicrographs was performed. Islets maintained on TCP or fibronectin for 7 days contained a large number of empty secretory granules (Figure 2A), while islets cultured on 3D-ECM contained significantly more dense-core secretory granules (with insulin) (Figure 2B). Moreover, the diameter of the dense-core secretory granules in islets cultured on 3D-ECM was greater than that found in islets cultured on either TCP or fibronectin (Figure 2C).

### 3.3 ∣ Islets cultured on 3D-ECM produced more insulin and were functionally more sensitive to glucose stimulation

**FIGURE 3:**
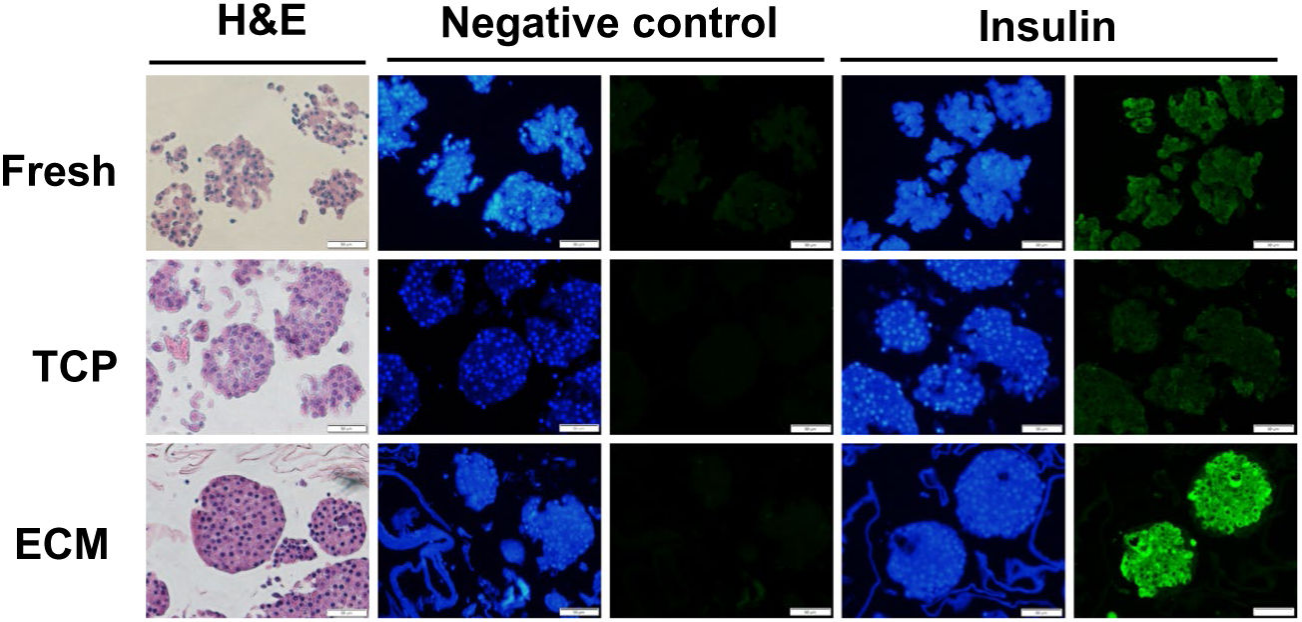
Islets cultured on 3D ECM were larger in size and had a more uniform smooth surface. Freshly isolated islets (Fresh) and islets cultured for 7 days on untreated TCP or TCP coated with 3D ECM were embedded, sectioned, and stained with H&E or prepared for immunofluorescence (IF) microscopy with an antibody against rat insulin (green fluorescence). Parallel sections were separately stained with a nonspecific isotype antibody as a negative control (Neg.) and DAPI to identify cell nuclei (blue fluorescence). Bar = 200 μm

**FIGURE 4:**
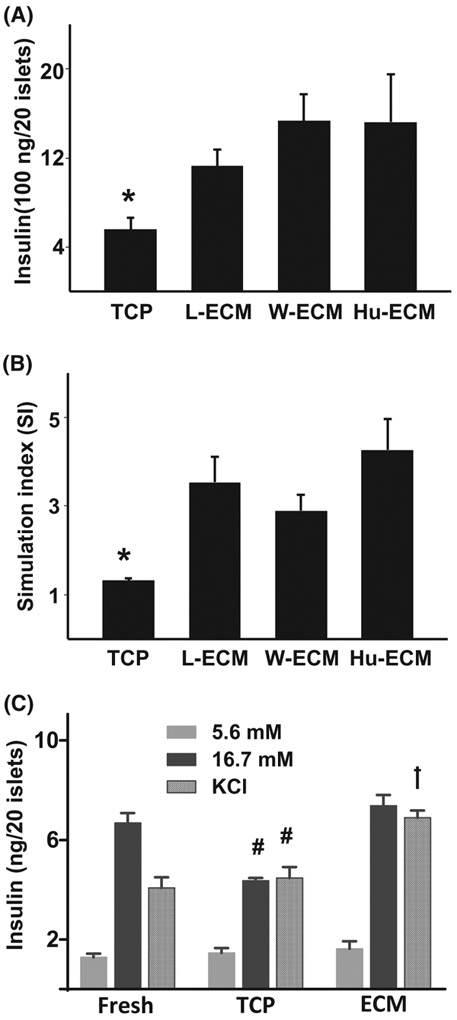
Islets cultured on 3D-ECM produced relatively more insulin and were functionally more sensitive to glucose stimulation. A, Total insulin produced by islets during 7 days in culture on untreated TCP or a 3D-ECM from various strains/species (*L-ECM*: Lewis rat ECM; *W-ECM*: Wistar-Furth rat ECM; *Hu-ECM*: human ECM) was determined using an ELISA specific for rat insulin. The data are the mean ± SEM, n = 5 replicates/treatment group (20 islets/replicate), *P < .01, versus all 3D ECM surfaces. B, Stimulation index of islets cultured for 7 days on untreated TCP or a 3D-ECM from various strains/species (see (A) above). For GSIS assay, islets were sequentially incubated in low glucose (5.6 mM) media for 60 minutes, followed by high glucose (16.7 mM) media for 60 minutes. Insulin release into the media was measured using an ELISA specific for rat insulin and a stimulation index calculated. The data are the mean ± SEM, n = 3 replicates/treatment group (20 islets/replicate). *P < .01, versus all 3D-ECM surfaces. C, GSIS assay was performed as in (B), except after treatment with 16.7 mM glucose for 60 minutes, islets were incubated a second time in 5.6 mM glucose for 60 minutes, and then, treated with 30 mM KCl for 60 minutes. Insulin release was measured as in (B). The data are the mean ± SEM, n = 6 replicates/treatment group (20 islets/replicate). ^#^*P* < .01, versus freshly isolated islets treated in an identical manner; ^†^*P* < 0.05, versus freshly isolated islets or islets cultured on TCP treated in an identical manner. Data were analyzed using one-way ANOVA with Bonferroni correction

Paraffin embedded sections of islets cultured on TCP or 3D-ECM and stained with H&E further confirmed that culture on 3D-ECM was superior to TCP as islets were larger in size and had a more uniform smooth surface. Further, some of the islets were in intimate contact with the surrounding matrix, implying better repair of the damage caused by the collagenase digestion step during isolation, which was not found with islets maintained on TCP (Figure 3). In addition, immunofluorescence (IF) microscopy, using an antibody to rat insulin and identical exposure settings for all specimens, showed that islets cultured on 3D-ECM exhibited brighter/more intense IF staining and less fusion than those cultured on TCP (Figure 3). The IF staining results were validated by measuring the total amount of insulin produced by islets cultured on TCP and 3D-ECMs (from two strains of rat and human donors) for 7 days (Figure 4A). To assess the functional capacity of islets cultured on the 3D-ECMs, a stimulation index was calculated based on the production of insulin in response to glucose treatment (Figure 4B). Indeed, rat islets maintained on the ECMs (irrespective of strain or species) responded to glucose stimulation in a significantly more robust manner than those on TCP (Figure 4B). Furthermore, to determine whether culture on the 3D-ECM restored β-cell membrane integrity, we compared the sensitivity of islets to potassium stimulation. Islets subjected to 5.6 mM glucose, followed by 16.7 mM glucose, and a return to 5.6 mM glucose were able to release a second round of insulin in response to treatment with 30 mM KCl, suggesting that culture on the 3D-ECM better maintained/restored the potassium channel in pancreatic β-cells than culture on TCP (Figure 4C). The data from this experiment also showed that islets cultured on 3D-ECM approached the level of insulin production of freshly isolated islets (Figure 4C).

### 3.4 ∣ Culture of islets on 3D-ECM restores vascular endothelial cells (VECs) and partial recovery of basement membrane-associated proteins on the islet surface

**FIGURE 5:**
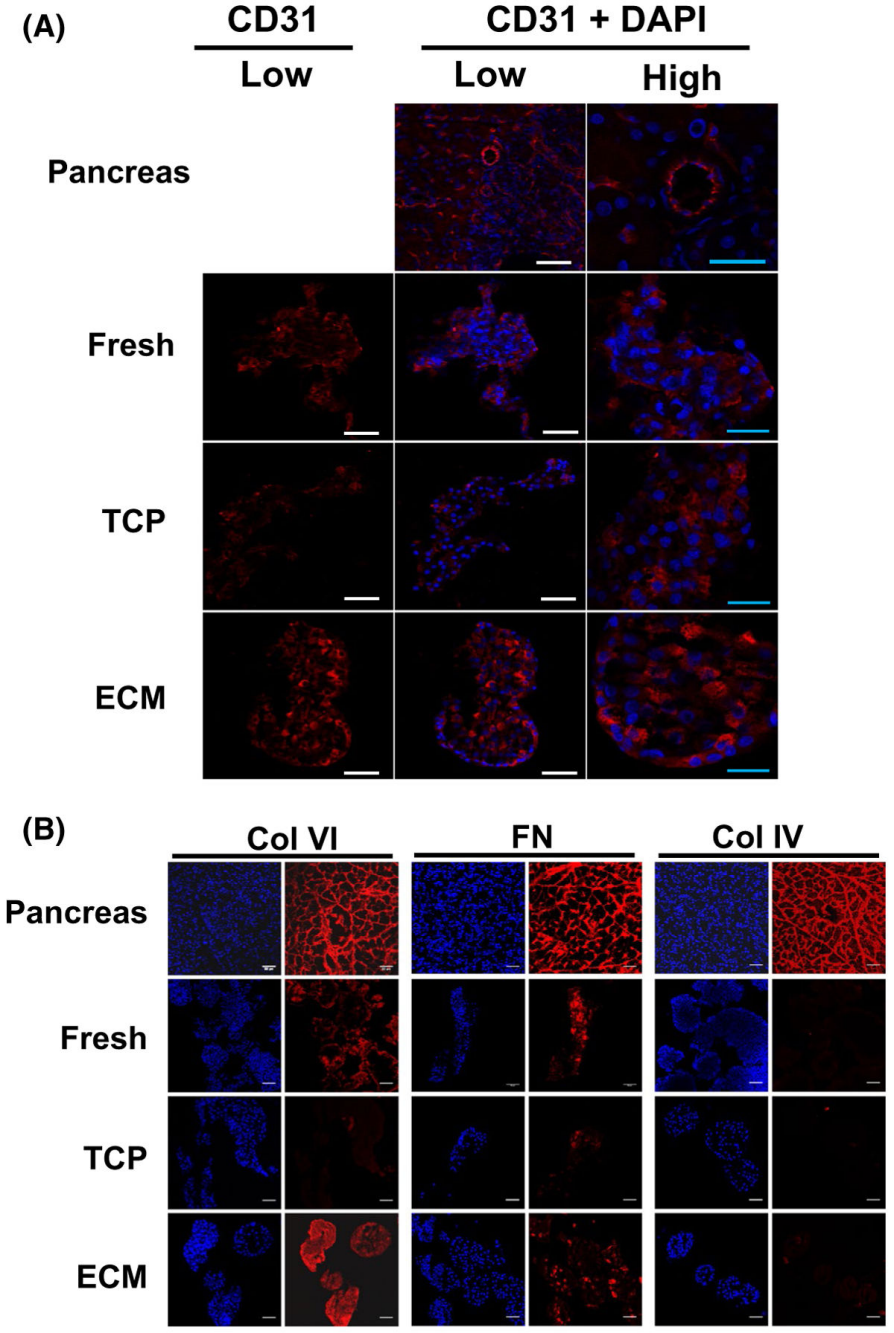
Culture of islets on 3D ECM restores VECs and partial recovery of basement membrane-associated proteins on the islet surface. A, VECs on the islet surface were stained for IF microscopy using an antibody to CD31 and viewed by confocal microscopy. Freshly isolated islets (Fresh) or islets cultured for 7 days on TCP or 3D ECM were examined. Rat pancreas served as a positive control; nuclei were counterstained with DAPI (blue). The white scale bar = 25 μm (ie, low magnification images), while the blue scale bar = 12.5 μm (ie, high magnification images). B, IF microscopy was used to identify the presence and location of fibronectin (FN), collagen IV (Col IV), and collagen VI (Col VI) in freshly isolated islets (Fresh), or islets cultured for 7 days on TCP or 3D ECM. Nuclei were counterstained with DAPI (blue). Bar = 25 μm

To further explore the apparent restoration of islet integrity and function with culture on 3D-ECM, we compared the presence of VECs and basement membrane-associated proteins on islets maintained on 3D-ECM versus TCP using IF staining (Figure 5). Islets cultured on ECM contained significantly more CD31-positive VECs, predominately located on the islet surface, than those that had been freshly isolated or cultured on TCP (Figure 5A). Moreover, collagen VI was found to stain more strongly in islets cultured on ECM, as compared to TCP, or freshly isolated (Figure 5B). Similarly, but to a lesser degree, fibronectin was detected in freshly isolated islets or islets cultured on 3D-ECM, but not TCP (Figure 5B). We were not able to detect collagen IV in any of the cultured islets, but positive IF staining was observed in pancreas tissue. In addition, we attempted and failed to detect laminin in any islet preparation (data not shown).

### 3.5 ∣ Preculture on 3D-ECM significantly attenuated islet immunogenicity and macrophage activation

**FIGURE 6:**
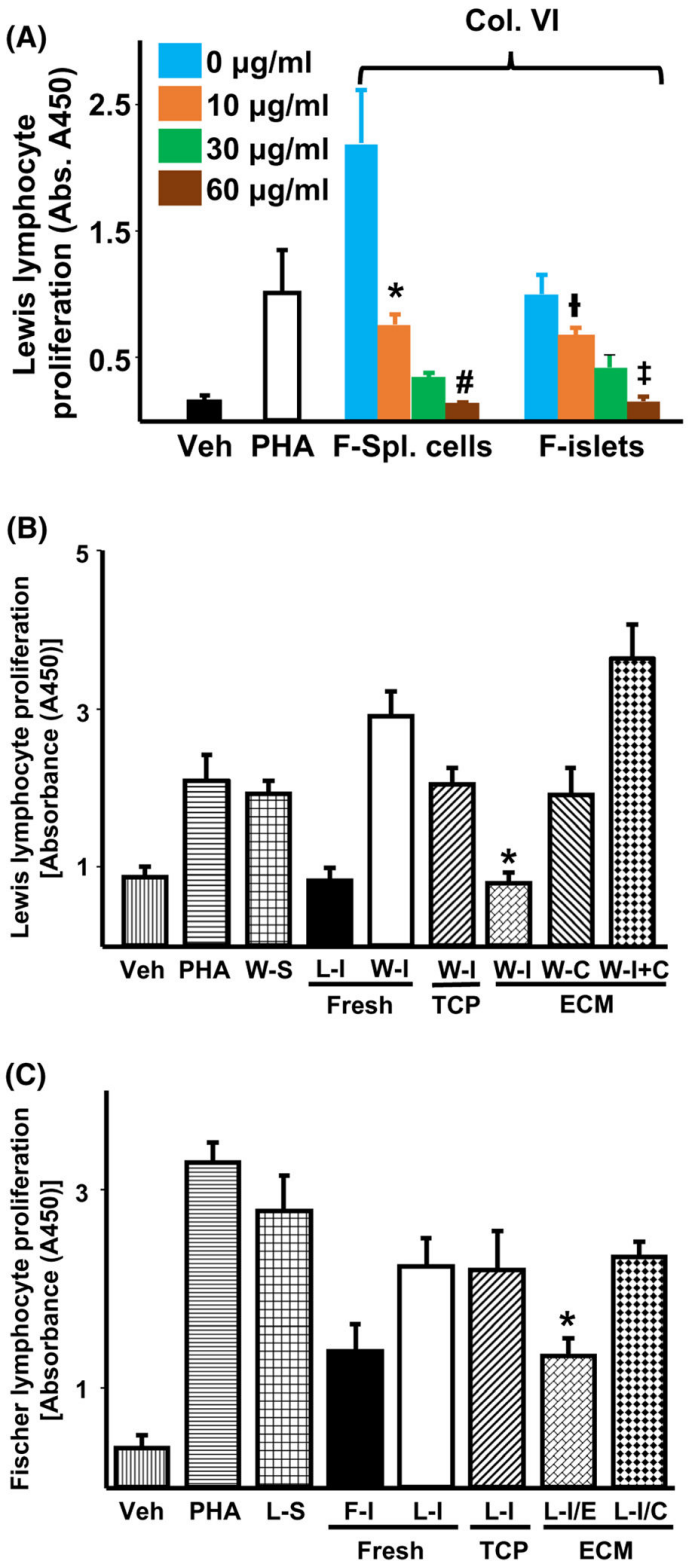
Preculture on 3D ECM significantly attenuated islet immunogenicity. A, Collagen VI (Col. VI) dose-dependently suppressed the proliferative response of Lewis splenocytes (lymphocytes) to coculture with Fischer splenocytes (*F-Spl cells*) or Fischer islets (*F-islets*). The data are the mean ± SEM, n = 4 replicates/treatment group, *P < .001, versus *F-Spl* cells without Col. VI; ^#^*P* < .001, versus *PHA* or *F-Spl* cells treated with 10 μg/mL Col. VI; ^†^*P* < .01, versus *PHA* or *F-islets* without Col. VI; and ^‡^*P* < .001, versus *PHA* or *F-islets* treated with 10 μg/mL Col. VI. Vehicle (*Veh*, negative control); and *PHA* (positive control). B, MLIC assay performed with Lewis lymphocytes mixed with freshly isolated islets (*Fresh*) or islets cultured for 7 days on TCP or 3D ECM W-S: Wistar-Furth splenocytes (positive control); *L-I/Fresh* and *W-I/Fresh*: Freshly isolated Lewis or Wistar-Furth islets; *W-I/TCP*: Wistar-Furth islets cultured for 7 days on TCP; *W-I/ECM*: Wistar-Furth islets cultured for 7 days on Lewis ECM; *W-C*: only “passenger” cells isolated from Wistar-Furth islets cultured for 7 days on Lewis ECM; and *W-I + C*: “passenger” cells added back to Wistar-Furth islets cultured for 7 days on Lewis ECM. The data are the mean ± SEM, n = 4 replicates/treatment group, *P < .05, versus other groups from WF rats. C, Effect of islet isolation method (EDTA vs collagenase) on MLIC assay performed with Fischer lymphocytes mixed with freshly isolated islets (*Fresh*) or islets cultured for 7 days on TCP or native ECM *L-S*: Lewis splenocytes (positive control); *F-I/Fresh and L-I/Fresh*: Freshly isolated Fischer or Lewis islets; *L-I/TCP*: Lewis islets cultured on TCP; *L-I/E/ECM*: Lewis islets cultured on ECM were detached by EDTA treatment; and *L-I/C/ECM*: Lewis islets cultured on ECM were detached by collagenase treatment. The data are the mean ± SEM, n = 3 replicates/treatment group, *P < .05, versus other Lewis groups. Data were analyzed using one-way ANOVA with Bonferroni correction

Next, we investigated whether fibronectin and Col. VI (basement membrane-associated proteins identified in our IF staining studies) were capable of suppressing the response of Lewis lymphocytes to Fischer islets or splenocytes using an MLIC assay. The results showed that exogenous collagen VI, but not fibronectin (data not shown), dose-dependently inhibited Lewis lymphocyte response to both Fischer spleen cells (*F-Spl. cells*) and freshly isolated Fischer islets (*F-islets*) (Figure 6A).

We then examined the immunogenicity of islets in an MLIC assay and found that islets from Wistar-Furth (WF) rats (*W-I*), cultured on native Lewis 3D-ECM, failed to stimulate Lewis lymphocyte proliferation (Figure 6B). In contrast, freshly isolated WF islets or WF islets cultured on TCP elicited a robust lymphocyte response that was just as strong as the PHA-treated (positive controls) or WF splenocyte experimental groups (Figure 6B). Interestingly, Lewis lymphocytes showed an increased response to WF islets, previously cultured on Lewis 3D-ECM, when mixed with “passenger” cells (*W-I+C*) detached from the ECM or the detached “passenger” cells alone (*W-C*), suggesting that “passenger” cells, contaminating the islet preparations, play a critical role in stimulating the lymphocyte response.

To further explore whether disruption of the peri-islet basement membrane, which occurs during collagenase digestion of donor tissue or after culture on TCP or native ECM, alters islet immunogenicity, we repeated the experiment described above (Figure 6B) and compared the release of islets with collagenase versus EDTA. We used Fischer lymphocytes in the MLIC assay to assess response to Lewis cells/islets. The results clearly showed that Lewis islets detached from the ECM by collagenase treatment (*L-I/C*) were significantly more stimulatory than those detached by EDTA treatment (*L-I/E*) (Figure 6C).

**FIGURE 7:**
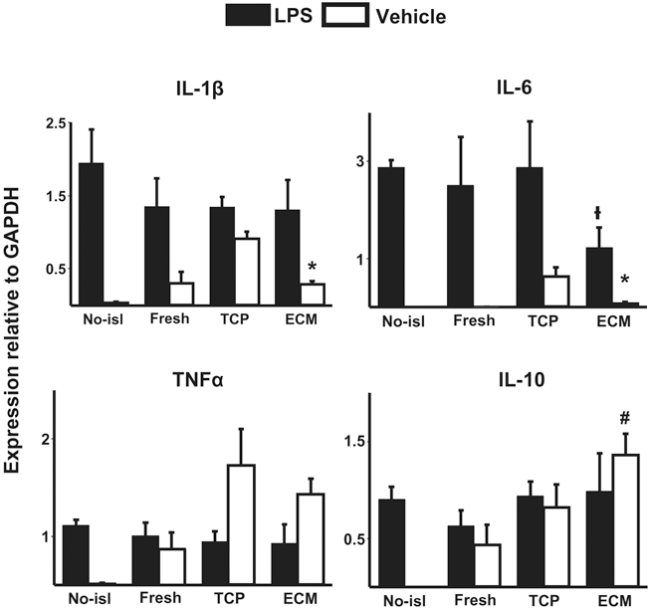
Coculture of human U937 macrophage-like cells with islets precultured on 3D-ECM not only attenuates the macrophage inflammatory response, but also increases the anti-inflammatory response as well. Human U937 cells were cocultured with freshly isolated islets (Fresh) or islets that had been cultured on TCP or 3D ECM for 7 days in the presence or absence (Vehicle) of LPS. After 48 hours of coculture, the macrophages were separated from the islets. Macrophage RNA was then extracted and analyzed for pro-inflammatory (IL-1β, IL-6, and TNF-α) and anti-inflammatory (IL-10) transcripts by real-time PCR. The data are the mean ± SEM, n = 3 replicates/treatment group. *P < .05, versus islets precultured on TCP in the absence of LPS; ^†^*P* < .05, versus no islets, freshly isolated islets, or islets precultured on TCP in the presence of LPS; and ^#^*P* < .05, versus no islets, freshly isolated islets, or islets precultured on TCP in the absence of LPS (=vehicle). Data were analyzed using one-way ANOVA with Bonferroni correction

Since macrophages are one of the major inflammatory cells recruited to the islet transplant site, we evaluated the influence of islets on macrophage activation by coculturing freshly isolated islets and islets previously precultured for 7 days on TCP or 3D-ECM for 48 hours with differentiated human macrophage-like U937 cells in the presence or absence of LPS (a known activator of macrophages) (Figure 7). Expression of the pro-inflammatory cytokine, IL-1β, by macrophages was significantly reduced only when islets had been precultured on 3D-ECM; similarly, pro-inflammatory IL-6 expression was reduced when islets were precultured on 3D-ECM in both the absence and presence of LPS, indicating the islets were capable of reducing IL-6 produced by LPS stimulated macrophages(Figure 7). Although not statistically significant, TNFα expression displayed the same trend as IL-1β in the absence of LPS. In contrast, expression of the anti-inflammatory cytokine, IL-10, was significantly increased when islets were precultured on 3D-ECM in the absence of LPS.

## 4 ∣ DISCUSSION

In the present study, we propose a new cell culture approach, previously described for the maintenance of MSCs,^12,13^ for preserving islet function and reducing islet immunogenicity prior to transplantation. The culture system is a native decellularized 3D-ECM containing many matrix components previously shown to play critical roles in islet survival, proliferation, and maintenance of function in vivo.^9,10,29^ The hypothesis of our study is that culture of rat pancreatic islets on 3D-ECM provides a unique microenvironment that promotes pancreatic islet attachment, viability, and maintenance of biological function, while attenuating immunogenicity.

We compared the adhesion of freshly isolated islets to TCP or TCP coated with fibronectin or 3D-ECM. We included fibronectin for comparison purposes because it has been implicated in promoting the retention of islet function.^30,31^ Our results suggest that the 3D-ECM enhances islet attachment and promotes cell survival (eg, fewer dead cells) (Figure 1). In contrast, in TCP cultures <20% of the islets attached and many islets (>60%) contained a considerable number of dead cells. Interestingly, cultures on the ECM contained more fibroblast-like cells in the vicinity of the islets, suggesting that the ECM retrieved “passenger” cells from the freshly isolated islets. It is known that these “passenger” cells express the major histocompatibility complex (MHC) class II antigen (in contrast to the islets) and are responsible for initiating an immune response from the host if they are not removed before transplantation.^8,32^ Our results showed that these fibroblast-like “passenger” cells, when detached from islet cultures, stimulated T cell proliferation (Figure 6B); furthermore, when islets were decontaminated of these “passenger” cells by preculture on ECM, significantly less T cell activation was observed.

We then analyzed the capacity of islets to produce insulin. Since the number, size, and content of insulin secretory granules is an excellent biomarker for assessing the physiological function of pancreatic beta-cells,^29^ we used TEM and found that the number and size of the “dense-core” insulin secretory granules in islets cultured on 3D-ECM were increased compared to TCP or fibronectin (Figure 2). Consistent with the TEM observations, islets stained with an insulin antibody for IF microscopy showed more intense staining with culture on 3D-ECM compared to TCP (Figure 3). These morphological results were confirmed by measuring the total amount of insulin released by islets maintained on TCP versus 3D-ECMs from donors of different strains/species. In each case, more insulin was produced by islets maintained on the ECMs (Figure 4A). Lastly, the glucose sensitivity of islets cultured on the three ECMs was significantly increased compared to TCP, based on stimulation index (Figure 4B). In addition, the sensitivity of islets cultured on 3D-ECM to potassium stimulation was also significantly enhanced compared to fresh islets or after culture on TCP (Figure 4C), further suggesting that culture on 3D-ECM better stabilized the K^+^ channel and maintained/restored the integrity of the β-cell membrane as compared to TCP.

It has been reported that islets contain VECs that play a major role in basement membrane formation and angiogenesis.^5,29^ Thus, we investigated whether the improvement in islet number and quality with culture on ECM was associated with a restoration of VECs. We observed more VECs distributed around the periphery of islets cultured on 3D-ECM compared to TCP (Figure 5A). The location of the VECs suggests that they contribute to the formation and maintenance of the basement membrane. To validate this possibility, we performed a series of IF microscopy studies using antibodies to basement membrane-associated proteins. The results demonstrated the presence of a remarkably high amount of collagen VI, moderate amounts of fibronectin, and little collagen IV and laminin (data not shown) in islets cultured on 3D-ECM (Figure 5B). In contrast, none of these proteins were detected in islets maintained on TCP. These findings suggest that the culture of islets on ECM promotes VEC recovery and partial restoration of the basement membrane and associated proteins, including collagen VI. Increasing evidence suggests that collagen VI is a critical architectural component of the cell niche and is associated with regeneration of muscle satellite and intestinal epithelial cells and with the repair of injured peripheral nerve.^33-35^ Indeed, it has been reported that collagen VI is a major component of the human islet ECM/niche and is present at more than two-times the amount of collagen types I and IV.^36^ More importantly, we found that collagen VI (but not fibronectin) dose-dependently inhibited the proliferation of Lewis T cells in response to both Fischer spleen cells and islets (Figure 6A), suggesting that collagen VI may play a critical role in protecting islets by reducing the immune response to the allografts.

Allogeneic islets precultured on 3D-ECM did not stimulate T cell proliferation in the MLIC assay. Interestingly, islets detached from the ECM by collagenase digestion, but not EDTA, lost their immunosuppressive properties and displayed the same stimulatory effect on Lewis lymphocyte proliferation as freshly isolated islets (Figure 6C), confirming that collagens, including collagen VI, are critically important to retaining low levels of islet immunogenicity. Initially, we expected that allogeneic islets would only lose their immunogenicity when cultured on ECM produced by cells of the same genetic background as the lymphocytes in the MLIC assay. In fact, we observed that loss of islet immunogenicity was only associated with islets cultured on ECM, irrespective of strain or specie. These findings suggest that at least two mechanisms are responsible for the decrease in immunogenicity observed with culture on 3D-ECM: (a) decontamination of “passenger” cells from freshly isolated islets; and (b) restoration of the basement membrane and associated proteins (eg, collagen VI).

Since macrophages are one of the inflammatory cells recruited to the islet transplant site, we evaluated the influence of islets on macrophage activation by coculturing freshly isolated islets and islets previously precultured for 7 days on TCP or 3D-ECM with human macrophage-like U937 cells in the presence or absence of LPS (a known activator of macrophages) (Figure 7). U937 cells displayed significantly less expression of pro-inflammatory cytokines, IL-1β and IL-6, a trend for lower expression of TNFα, but a high level of the anti-inflammatory cytokine, IL-10, when cocultured with islets precultured on 3D-ECM (vs TCP) (Figure 7). This implies that the macrophage response to islets precultured on ECM, as compared to TCP, favored expression of the M2 (anti-inflammatory) phenotype. Taken together with our studies demonstrating decreased immunogenicity, the data suggest that culture of islets on ECM may attenuate both the inflammatory activity and immune response.

In summary, the results demonstrate that our unique 3D-ECM culture system, which mimics the pancreatic niche ex vivo, promotes islet attachment, growth, and function. This culture system efficiently decontaminates “passenger” cells from freshly isolated islets and restores the basement membrane and associated proteins (eg, collagen VI) that are essential for reducing islet immunogenicity. If these results are further validated in an appropriate in vivo animal model, and then, translated to the clinic, culture of islets on 3D-ECM will provide a method for overcoming two major issues, which have hindered islet transplantation therapy: (a) the poor yield of islets from donated pancreas tissue and (b) the need for life-long immunosuppression.

## Abbreviations:

3D ECM: native three dimensional bone marrow-derived ECM
AO: acridine orange
BM: bone marrow
BM-MSCs: bone marrow-derived mesenchymal stem cells
BrdU: bromodeoxyuridine
ECM: extracellular matrix
FBS: fetal bovine serum
GAPDH: glyceraldehyde-3-phosphate dehydrogenase
GSIS: glucose-stimulated insulin secretion
IEQ: islet equivalents
IF: immunofluorescence
LPS: lipopolysaccharide
MLIC: assay, mixed lymphocyte-islet coculture assay
MSCs: mesenchymal stem cells
PBS: phosphate-buffered saline
PHA: phytohemagglutinin
PMA: phorbol-12-myristate-13-acetate
PI: propidium iodide
T1D: type 1 diabetes
TCP: tissue culture plastic/polystyrene
TEM: transmission electron microscopy
VECs: vascular endothelial cells
VEGF: vascular endothelial growth factor
